# Our New York Letter

**Published:** 1886-01

**Authors:** Wm. Penny

**Affiliations:** New York


### OUR NEW YORK LETTER.

NEW YORK, Oct. 17, 1885.

TO THE EDITOR OF DANIEL'S TEXAS MEDICAL JOURNAL:

*Dear Sir:—*

THINKING you would like a few notes on Surgery in the great center, and as I make my headquarters at the Poly-clinic, I will give you a brief report of a few cases as illustrative of the methods in vogue here.

Dr. Wyeth I have seen in a number of operations in the last week. He is characterized by his extreme carefulness and the anatomical accuracy of his incisions in a case of ulcer of the tongue, diagnosed to be cancerous, on which I witnessed him operate. He commenced by the extremely tedious and difficult operation of ligating the lingual artery, tying every little artery as he advanced his incisions. He ligated the artery with a loss of less than an ounce of blood. The subsequent operation of removing a large wedge-shaped piece from the side of the tongue was bloodless.

In the treatment of all surgical wounds here, antiseptic measures are taken, and so great is their confidence in the efficacy of these means, that a surgeon, recently, in a thigh amputation, promised his class to bring the case before them one week from that day, with complete union; and while we might admit the possibility of this result under the old treatment, no surgeon would have risked his reputation on such a prediction.

Strictures of the urethra are plentiful everywhere in the civilized world, but they drift into the hospitals and dispensaries of the large cities in numbers that would astonish the average country doctor. The treatment here consists of cutting either internally or externally. For the first-named operation, some modification of Otis,urethretome is used almost exclusively. Dr. Wyeth usually has one or two cases at his clinic every week. These cases are looked on as rather among the minor than the major surgical operations. I had the pleasure of witnessing Dr. Wyeth operate for external perineal urethrotomy, cutting down on a filiform bougie. Now, while it is not an extremely difficult operation to find a filiform bougie, it requires a good deal of manual dexterity to do it without having to hunt with the point of a knife or a probe for the guide.

In operating for hydrocele, something that we all have to do occasionally, Dr. Wyeth draws off the fluid with a small trocar and then injects about fifteen drops of pure carbolic acid into the sac, withdraws the canula, kneads the sac, and allows the whole of the acid to remain. I witnessed this small operation as performed by the doctor. He says that he has never had any bad results, and is successful in nearly all of his cases.

In orthopedics, that grand old man, Sayre, still holds his court at Belleview. He is a little inclined to be profane at times, but he inspires a great deal of enthusiasm in his class; while that polished gentleman, Gibney, holds forth at the Polyclinic. From his long connection with the Hospital for Ruptured and Crippled, and his marked ability, he attracts a large amount of clinical material.

Regret that I have not time to give you some jottings on the lectures here; may at some other time.

Yours, WM. PENNY, M. D.

